# Plant stoichiometric responses to elevated CO_2_ vary with nitrogen and phosphorus inputs: Evidence from a global-scale meta-analysis

**DOI:** 10.1038/srep18225

**Published:** 2015-12-14

**Authors:** Wenjuan Huang, Benjamin Z. Houlton, Alison R. Marklein, Juxiu Liu, Guoyi Zhou

**Affiliations:** 1 Key Laboratory of Vegetation Restoration and Management of Degraded Ecosystems, South China Botanical Garden, Chinese Academy of Sciences, Guangzhou 510650 China; 2 Department of Land, Air, and Water Resources, University of California-Davis, One Shields Avenue, Davis, CA 95616 USA

## Abstract

Rising levels of atmospheric CO_2_ have been implicated in changes in the nitrogen (N) and phosphorus (P) content of terrestrial vegetation; however, questions remain over the role of C, N and P interactions in driving plant nutrient stoichiometry, particularly whether N and P additions alter vegetation responses to CO_2_ enrichment singly. Here we use meta-analysis of 46 published studies to investigate the response of plant N and P to elevated CO_2_ alone and in combination with nutrient (N and P) additions across temperate vs. tropical biomes. Elevated CO_2_ reduces plant N concentrations more than plant P concentrations in total biomass pools, resulting in a significant decline in vegetation N/P. However, elevated CO_2_ treatments in combination with N additions increase plant P concentrations, whereas P additions have no statistical effect on plant N concentrations under CO_2_ enrichment. These results point to compensatory but asymmetrical interactions between N, P and CO_2_; that changes in N rapidly alter the availability of P, but not the converse, in response to increased CO_2_. Our finding implies widespread N limitation with increasing atmospheric CO_2_ concentrations alone. We also suggest that increased anthropogenic N deposition inputs could enhance plant N and P in a progressively CO_2_-enriched biosphere.

Atmospheric carbon dioxide concentrations (CO_2_) have grown considerably due to human actions, with an anticipated peak concentration of greater than 700 μmol mol^−1^ by the end of this century^1^. The modern rise in CO_2_ is thought to stimulate terrestrial productivity^2^, resulting in negative feedback on atmospheric CO_2_ levels and reductions in the pace of climate warming; however, the magnitude of this feedback is thought to be constrained by growth-limiting nutrients, especially nitrogen (N) and phosphorus (P) availability^3^,^4^,^5^. Here we use meta-analysis to examine changes in N, P and N/P in plants in response to elevated CO_2_ and nutrient fertilization across a range of sites and conditions.

The stoichiometry of plant N and P concentrations has provided insight in patterns of N versus P limitation across terrestrial ecosystems^6^,^7^,^8^, including nutrient limitation responses to elevated CO_2_^9^. Past work has demonstrated that elevated CO_2_ reduces plant N concentrations generally^10^,^11^, largely as a result of the carbohydrate dilution^12^ and the inhibition of nitrate assimilation within plants^13^. The effect of CO_2_ enrichment on plant P concentrations has been more variable than for N, with evidence for decreased^14^, increased^15^ or neutral effects on plant P^16^ in individual study systems. Using meta-analysis, Duval *et al.*^17^ showed that plant P responses to elevated CO_2_ varied among plant functional groups. More recently, Yuan and Chen^18^ showed that elevated CO_2_ on average decreases plant N/P across an array of sites; however, this study did not examine tropical plant N/P responses, despite tropical systems having different nutrient conditions than many temperate ones^3^. Further, past syntheses have not addressed the response of plant N/P to CO_2_ in combination with other N, P or N plus P additions. Questions remain over whether N or P will become progressively more limiting under elevated CO_2_, and how such limitations will be affected by changes in P and N inputs across temperate vs. tropical ecosystems. This is critical given that C, N and P are among the most anthropogenically altered biogeochemical cycles on Earth^19^.

It is increasingly important to recognize that plant responses to CO_2_ are acting in combination with other global changes, particularly changes in anthropogenic N deposition and P inputs through cropland fertilization, which can also affect patterns of nutrient cycling and limitation on land. Nitrogen inputs can increase plant productivity and lead to over-enrichment with N^20^, and can even result in what has been termed “anthropogenic P limitation”^3^–the progressive occurrence of P limitation to plant productivity via chronic N inputs from human sources. Indeed, past evidence indicates increased plant N/P with increased N inputs^18^,^21^. On the other hand, the increasing use of P fertilizer is improving its availability in ecosystems^4^. The response of plant N/P to altered P availability can be expected, showing a decrease in plant N/P under P fertilization^18^.

Yuan and Chen^18^ suggested that multiple global change treatments including elevated CO_2_, precipitation, warming and N deposition result in additive effects on plant N/P ratios. However, they did not study the effects of elevated CO_2_ with P additions or with N and P additions combination. Evidence for widespread nutrient co-limitation suggests that the cycling of one nutrient can influence the availability of another^22^. Past work in Hawaii has shown that N fertilization increased plant-root and soil phosphatase levels^23^, a finding that was later demonstrated across a wide variety of terrestrial ecosystems^24^. P fertilization was found to enhance N uptake by plants and make plant roots competitive for N against free-living microbes in forest ecosystems^25^. These interactions between N and P have also been shown to play a role in global patterns of symbiotic N_2_-fixation^26^, and allow plants and mycorrhizal fungi to allocate biomass and energy towards the acquisition of limiting resources in general^27^.

Here, we analyze plant N/P in response to elevated CO_2_ alone and in combination with N, P and N + P additions to test the hypotheses that (1) elevated CO_2_ decreases plant N and P concentrations;(2) nutrient inputs prevent declines in plant nutrient contents of the fertilized nutrient under elevated CO_2_; and (3) inputs of N or P alters plant nutrient contents of the other nutrient under elevated CO_2_ due to compensatory interactions among C, N and P cycles.

## Results

**Figure 1:**
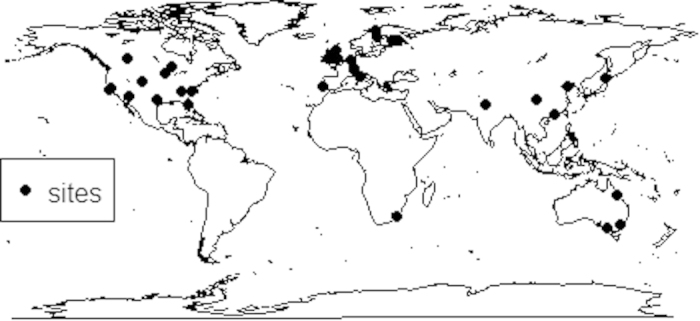
Map of sites of the collected studies. Figure 1 was created by R (R Core Team, 2014)^54^ using the maps package.

We collected 133 observations from 46 separate studies to examine CO_2_ treatment effects on plant N/P (see Supplementary References). The majority of the data was from the temperate regions (70%) as opposed to the (sub-)tropics (28%) (see Fig. 1 and Supplementary Table S1). Of these observations, 28 measured plant N/P in FACE; 97 measured plant N/P in chambers; and the remaining 8 were classified as others including branch bag techniques, natural CO_2_ springs and screen-aided CO_2_ control. Of these species, 86 observations were collected from woody plants and 47 from non-woody plants. There were 19 observations for legumes and 114 for non-legumes. Most studies focused on aboveground plant nutrient concentrations (102 observations) compared to 27 belowground observations and 4 whole-plant concentrations reported. Of the compiled studies, there were 34 observations for elevated CO_2_ with N alone, 22 observations for elevated CO_2_ with added P alone, and 15 observations for elevated CO_2_ with added N and P in combination (see Supplementary Table S1).

### Effects of elevated CO_2_ on plant N, P and N/P

**Figure 2:**
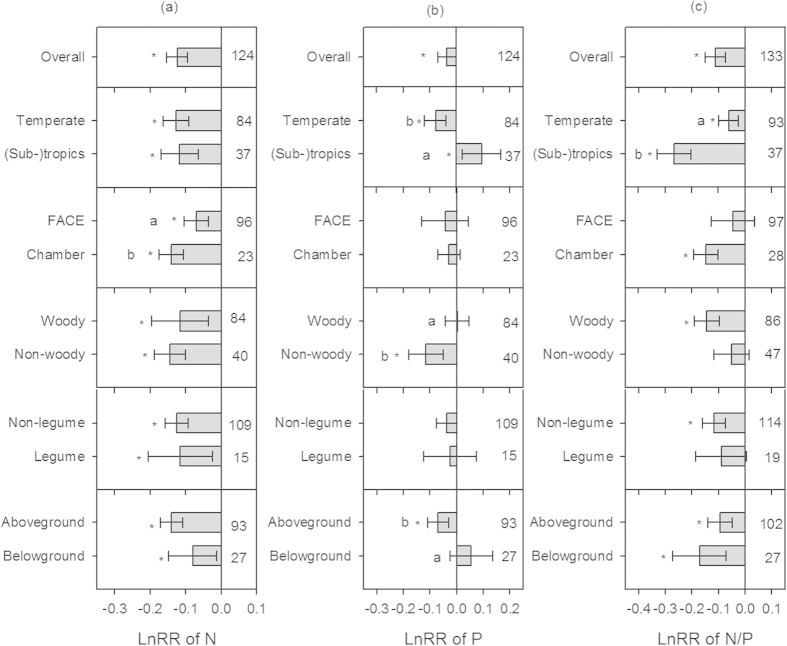
Effects of elevated CO_2_ on plant N and P stoichiometry. (**a**) plant N concentrations; (**b**) plant P concentrations; (**c**) plant N/P. The error bars show the 95% confidence interval of LnRR. LnRR, the natural logarithm of response ratio that is calculated as the experimental mean divided by the control mean. The asterisk (*) denotes the effect of elevated CO_2_ was significant. Different lowercases in the left indicate significant differences between groups. The number of observations for each category is given in the right.

Plant N concentrations decreased significantly with elevated CO_2_ (~12%), regardless of the climatic zones, the kind of CO_2_ delivery, plant functional group or plant tissue examined (Fig. 2a). Across all observations in this analysis, elevated CO_2_ was associated with an averaged 4% decrease in plant P concentrations, but not consistently among factors as observed for decreased N concentrations (Fig. 2b). Rather, elevated CO_2_ decreased plant P concentration in temperate regions but increased it in (sub-)tropics. CO_2_-incuced declines in plant P concentrations were observed for non-woody plants and aboveground tissues, but not for woody plants and belowground tissues. Hence, elevated CO_2_ significantly decreased plant N/P by 11% compared to control conditions, but this response varied with climatic zones (Fig. 2c). Overall, elevated CO_2_ decreased plant N/P to a greater extent in (sub-)tropics (23%) than in temperate regions (6%). CO_2_ enrichment caused significant declines in plant N/P for chambers (14%) but not FACE experiments, for woody species (13%) but not non-woody species, and for legumes (11%) but not for non-legumes.

### Effects of elevated CO_2_ with nutrient fertilization on plant N, P and N/P

**Figure 3:**
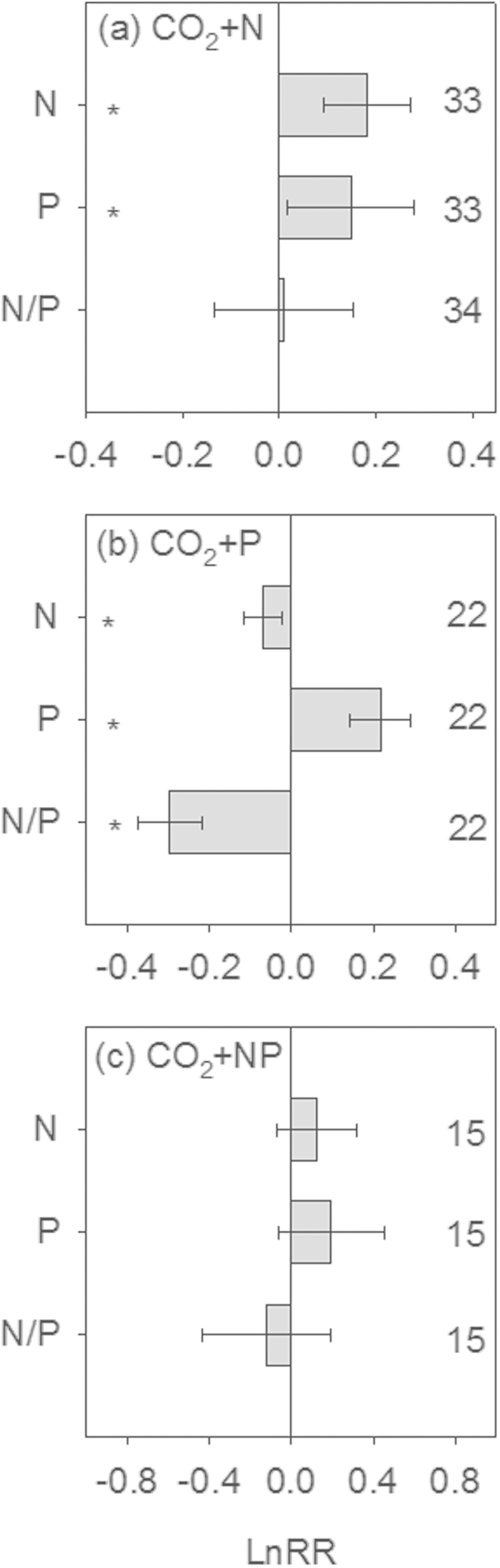
Effects of elevated CO_2_ and nutrient fertilization on plant N and P stoichiometry. (**a**) elevated CO_2_ with N fertilization (CO_2_ + N); (**b**) elevated CO_2_ with P fertilization (CO_2_ + P); (**c**) elevated CO_2_ with N and P fertilizations (CO_2_ + NP). The error bars show the 95% confidence interval of LnRR. LnRR, the natural logarithm of response ratio that is calculated as the experimental mean divided by the control mean. The asterisk (*) denotes the effect of treatments was significant. The number of observations for each category is given in the right.

In the dataset of elevated CO_2_ with N fertilization, elevated CO_2_ alone (without N fertilization) significantly decreased plant N concentration (3%) and increased plant P concentration (16%), thus resulting in a decrease of 20% in plant N/P (see Supplementary Fig. S1a). However, elevated CO_2_ with N addition did not affect plant N/P, as it led to statistically equivalent increases in plant N concentration (20%) and in plant P concentration (16%) (Fig. 3a). The response ratios of plant N concentrations, P concentrations and N/P with CO_2_ enrichment and N fertilization were not related to the amount of N added (see Supplementary Fig. S2).

Elevated CO_2_ without P fertilization tended to negatively affect plant N concentrations, P concentrations and N/P (see Supplementary Fig. S1b). However, the patterns were altered when P was added. CO_2_ plus P treatments consistently decreased plant N concentrations (7%), increased plant P concentrations (24%) and decreased plant N/P ratios (26%) (Fig. 3b). The responses of plant N and P concentrations and plant N/P to elevated CO_2_ with P fertilization were not related to the amount of P added (see Supplementary Fig. S2).

In the dataset of elevated CO_2_ with N and P fertilizations, N and P fertilizations did not significantly affect plant N concentration, P concentration and N/P responses to elevated CO_2_ (see Supplementary Fig. S1c and Fig. 3c).

### Relationships between plant N and P concentrations

**Figure 4:**
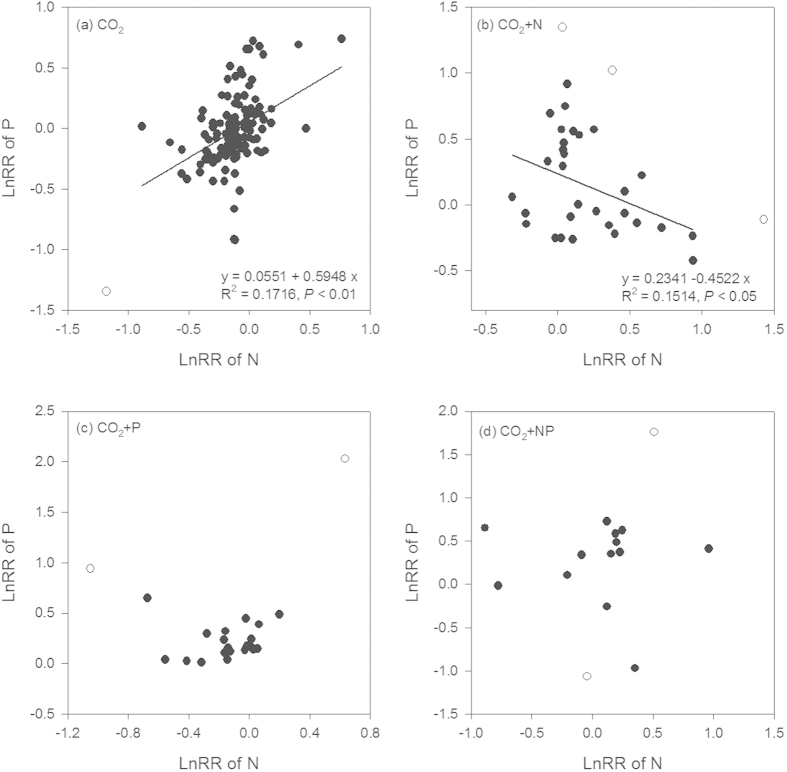
Relationships between the response ratios of plant N and P concentrations to elevated CO_2_ alone and elevated CO_2_ with nutrient fertilization. LnRR, the natural logarithm of response ratio that is calculated as the experimental mean divided by the control mean. The circles indicate the values which were greater than 1 or smaller than −1. Linear regressions were estimated using the points excluding the circles. (**a**) Elevated CO_2_ alone (CO_2_); (**b**) elevated CO_2_ with N fertilization (CO_2_ + N); (**c**) elevated CO_2_ with P fertilization (CO_2_ + P); (**d**) elevated CO_2_ with N and P fertilizations (CO_2_ + NP).

The response ratios (the experimental mean divided by the control mean) of plant N concentrations to elevated CO_2_ were significantly positively related to those of plant P concentrations (R^2^ = 0.1716, *P* < 0.01) (Fig. 4a). There was a significant negative relationship between the response ratios of plant N and P concentration under elevated CO_2_ when N added (Fig. 4b), while no significant relationship was observed under elevated CO_2_ when P or NP added (Fig. 4c,d).

## Discussion

This cross-system analysis supports expectations for our first hypothesis – that plant N and P contents decline systematically under CO_2_ enrichment singly. This result was clear and systematic in the case of plant N concentrations and generally confirmed results of previous research^10^,^11^,^28^,^29^. In contrast, plant P responses to CO_2_ have been less-examined. Our results showed that P exhibited a more complex CO_2_ response than N across a broad range of terrestrial ecosystems, which resulted in some differences in plan N/P. For example, plant P was increased under elevated CO_2_ in (sub-)tropics rather than temperate regions, which imply that relatively high soil N availability in (sub-)tropics could help plants acquire P with high energy (C) inputs^26^. While aboveground P concentrations declined with CO_2_ enrichment, P in belowground tissues did not change with elevated CO_2_. This suggests that plants may be allocating more P to roots under elevated CO_2_, or actively mining soil P pools, perhaps via increased investment in mycorrhizal fungi and fine roots^30^,^31^. In addition, woody plants showed almost no change in P concentrations under elevated CO_2_, whereas P concentrations of herbaceous vegetation declined significantly, revealing strong functional-group dependencies in plant P responses. The relative stability in legume N/P under elevated CO_2_ indicate that N_2_-fixing species has the ability to balance N and P through direct access to N from atmosphere and investment in phosphatase and mycorrhizae to acquire P^26^,^32^.

Our results showed net declines in plant N/P under elevated CO_2_, which were in line with those reported for temperate ecosystems by Yuan and Chen^18^. Our study advances their analysis by revealing that this decline was driven by much larger reductions in plant N concentrations (12%) than plant P concentrations (4%), including both temperate and tropical ecosystems. In fact, the coefficient of the linear regression (slope) of the responses of plant P vs. N contents to elevated CO_2_ was less than unity (*P* < 0.01) (Fig. 4a). This means that the changes in plant N contents exceeded those of plant P across our global data synthesis. These findings are consistent with a recent study of C3 plants in which a 15% reduction in N but only 9% reduction in P occurred with enhanced CO_2_ concentrations^33^.

Generally, the decline in plant N/P we observed points to more substantial N vs. P limitation with CO_2_ enrichment, which can explained by factors that differentially affect plant metabolism of N vs. P^11^,^12^. Elevated CO_2_ may increase the efficiency of photosynthesis or metabolically down-regulate photosynthetic enzymes, thus causing increasing photosynthetic N use efficiency with decreasing N supplies^34^,^35^. Further, higher ATP requirements in response to elevated CO_2_ would disproportionally increase P vs. N demands in ecosystems^36^,^37^. In addition, elevated CO_2_ could increase plant growth rates, which requires P-rich ribosomal RNA (rRNA)^38^,^39^. Any combination of these mechanisms would lower N/P in response to elevated CO_2_.

Our results also support the second hypothesis – that nutrient inputs alter plant N/P response to elevated CO_2_ (Fig. 4). This result implies that plant nutrient responses to rising CO_2_ will depend on the magnitude of changes in N and P inputs in ecosystems^9^. This is important given that to anthropogenic alterations of N and P that have occurred in concert with atmospheric CO_2_. The positive responses of plant N and P to N and P inputs, respectively, have the potential to compensate for their declines induced by elevated CO_2_. These results contrast with Cotrufo *et al.*^10^, who found no evidence for an effect of N fertilization on tissue N concentrations beyond the declines observed with elevated CO_2_. This discrepancy could reflect our differing approaches; Cotrufo *et al.*^10^ divided their observations into arbitrary classes of N addition whereas we used a combination of meta-analysis and regression to examine nutrient by CO_2_ effects. Elevated CO_2_ frequently stimulates rates of photosynthesis, yet N or P supply can modulate the magnitude of the CO_2_-fertilization effect via its effect on the carboxylation capacity^29^,^40^,^41^. Wholesale reductions or longer-term acclimation of photosynthesis to elevated CO_2_ can be associated with declines in tissue N concentrations^42^,^43^. Our results imply that either N or P supplies can offset any down-regulation of photosynthesis in response to CO_2_ enrichment.

Finally, we found support for the third hypothesis – that input of N or P alters plant nutrient contents of the other nutrient– in the case of N by CO_2_ additions but not for P by CO_2_ treatments. Plant P concentrations increased under elevated CO_2_ with N addition, thereby stabilizing plant N/P across terrestrial ecosystems. Nitrogen additions have been shown to enhance P uptake in roots from P-deficient soluble sources under elevated CO_2_ by inducing a set of morphological, physiological, and molecular adaptive strategies^44^. Numerous studies have shown that N additions can facilitate P acquisition via alterations in root development^45^ and rhizosphere pH^46^, and via organism investments in phosphatase enzymes^24^. However, there was no apparent trade-off in responses of plant N and P to elevated CO_2_ or N addition in our meta-analysis. On the contrary, we found no evidence for a compensating effect of CO_2_ plus P fertilization treatments on the declines in plant N concentrations under CO_2_. Previous analysis on the effect of P additions on plant N concentrations have been inconsistent, with no changes in plant N observed under N-rich conditions^47^, decreases observed in N-poor soils^48^, and increases under conditions of P limitation^25^. Our results suggest that, on average, P additions do not alter the pattern of systematically declining plant N concentrations with CO_2_ enrichment, consistent with several past studies from individual sites^49^,^50^.

In summary, our extensive meta-analysis shows that elevated CO_2_ decreases plant N/P, implying a tendency for N rather than P limitation of terrestrial productivity to arise in response to elevated CO_2_ alone. However, when CO_2_ and N increase in concert, as is occurring over much of the terrestrial biosphere, compensatory interactions among C, N and P cycles stabilize total plant N/P. These results suggest that, ecosystems exposed to elevated CO_2_ and N deposition will not necessarily progress rapidly into conditions of P limitation, owing to a suite of non-symmetrical interactions between N and P. Longer-term effects remain unclear, however, models used to forecast the N and P cycles in response to CO_2_ and climate change would be well-served to consider such compensatory interactions in determining the capacity for additional CO_2_ sequestration in the future

## Methods

We searched ISI Web of Science, using the terms “elevated CO_2_”, “CO_2_ enrichment”, and “CO_2_ enriched”, in combination with “nutrient”, “nitrogen” and “phosphorus”, to create a database for our meta-analysis. The data were restricted to studies performed in natural terrestrial ecosystems, i.e., excluding agricultural or other managed ecosystems. All studies included in this analysis measured N/P or N and P concentrations (from which we could calculate N/P) under ambient CO_2_ and elevated CO_2_. We also limited our data to studies where means, standard deviations of the mean, and number of replicates were reported or could be calculated. For studies that measured N and P concentrations at multiple time-points, only the final value was used to maintain the statistically independence between individual observations. DataThief^51^ was used to acquire numbers from figures where data were not presented in tables. The units for N and P concentrations were all converted to mg g^−1^. All N/P are presented here on a mass basis.

We categorized the data by climatic zones, experiment types, plant characteristics, and presence any kind of nutrient fertilization involving N and P. The climatic zones included arctic, temperate and (sub-)tropical zones. Research facilities used to increase CO_2_ concentration were classified as Free-Air Carbon dioxide Enrichment (FACE) and chambers; plants were categorized based on functional type (woody vs. non-woody plants, and non-legume vs. legume) and plant tissues examined (i.e., aboveground vs. belowground).

To examine interactions between elevated CO_2_ and nutrient (N and P) fertilization, we also collected data on the level N and/or P additions where available, including elevated CO_2_ with N fertilization (from 1 g N m^−2^ yr^−1^ to 20 g N m^−2^ yr^−1^) (CO_2_ + N), elevated CO_2_ with P fertilization (from 1.5 g P m^−2^ yr^−1^ to 16 g P m^−2^ yr^−1^) (CO_2_ + P), and elevated CO_2_ with N and P fertilizations (CO_2_ + NP). For each of the three datasets, we made two comparisons: elevated CO_2_ without nutrient fertilization vs. control (ambient CO_2_ without nutrient fertilization) and elevated CO_2_ with nutrient fertilization vs. control.

The treatment effects were examined by calculating response ratios (RR) from each individual study. RR is used as an indicator of effect size, and is calculated as the experimental mean divided by the control mean (ambient CO_2_ without nutrient fertilization)^52^. The natural logarithm of response ratio (LnRR) was then used to perform statistical analysis as it equally weighs the negative and positive responses^52^. The mean effect size was calculated using a mixed-effects model of the meta-analytical software MetaWin 2.0^53^. The 95% confidence intervals (CI) for effect-size estimates were calculated using resampling techniques. The effects of experimental treatments of a variable were considered to be significant if the 95% CI values did not overlap with zero^52^. We compared the responses to experimental treatments between groups in each category, in which each group should have at least five studies^52^. The responses to experimental treatments among groups in each category were considered to be significantly different when the between-group heterogeneity was significant (*P* < 0.05)^52^. The differences between means of the groups were considered statistically significant if their 95% CI values did not overlap.

## Additional Information

**How to cite this article**: Huang, W. *et al.* Plant stoichiometric responses to elevated CO_2_ vary with nitrogen and phosphorus inputs: Evidence from a global-scale meta-analysis. *Sci. Rep.* **5**, 18225; doi: 10.1038/srep18225 (2015).

## Supplementary Material

Supplementary Information
