# Supplementary material for: Plant stoichiometric responses to elevated CO2 vary with nitrogen and phosphorus inputs: Evidence from a global-scale meta-analysis
Source: Sci Rep. 2015 Dec 14;5:18225. doi: 10.1038/srep18225 (PMC4677399; doi:10.1038/srep18225)
Supplement: Supplementary Information [file srep18225-s1.pdf]

Plant stoichiometric responses to elevated CO_2 vary with nitrogen and phosphorus inputs: Evidence from a global-scale meta-analysis

Wenjuan Huang, Benjamin Z. Houlton, Alison R. Marklein, Juxiu Liu & Guoyi Zhou

Table S1. Sample sizes of the collected studies

		N	P	N/P
Elevated CO_2				
	Total	124	124	133
Climatic zone	Temperate	84	84	93
	(Sub-)tropics	37	37	37
	Subarctic	3	3	3
Experiment type	FACE	23	23	28
	Chamber	96	96	97
	Branch bags	2	2	2
	Natural CO_2 springs	3	3	3
	Screen-Aided CO_2 control	0	0	3
Plant type	Woody plants	84	84	86
	Non-woody plants	40	40	47
	Non-legume	109	109	114
	Legume	15	15	19
Plant tissue	Aboveground	93	93	102
	Belowground	27	27	27
	Whole plants	4	4	4
Elevated CO_2 with N		33	34	34
Elevated CO_2 with P		22	22	22
Elevated CO_2 with NP		15	15	15

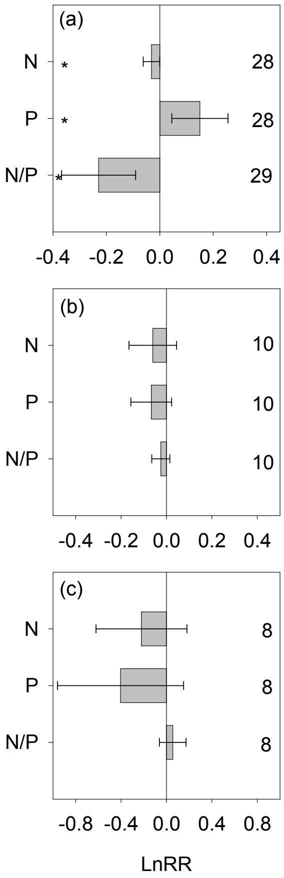

Figure S1. Effects of elevated CO_2 on plant N and P stoichiometry in the dataset of elevated CO_2 with nutrient fertilization. (a) dataset of elevated CO_2 with N fertilization; (b) dataset of elevated with P fertilization; (c) dataset of elevated CO_2 with N and P fertilizations. LnRR, the natural logarithm of response ratio that is calculated as the experimental mean divided by the control mean. The error bars show the 95% confidence interval of LnRR. The asterisk (*) denotes the effect of treatments was significant. The number of observations for each category is given in the right.

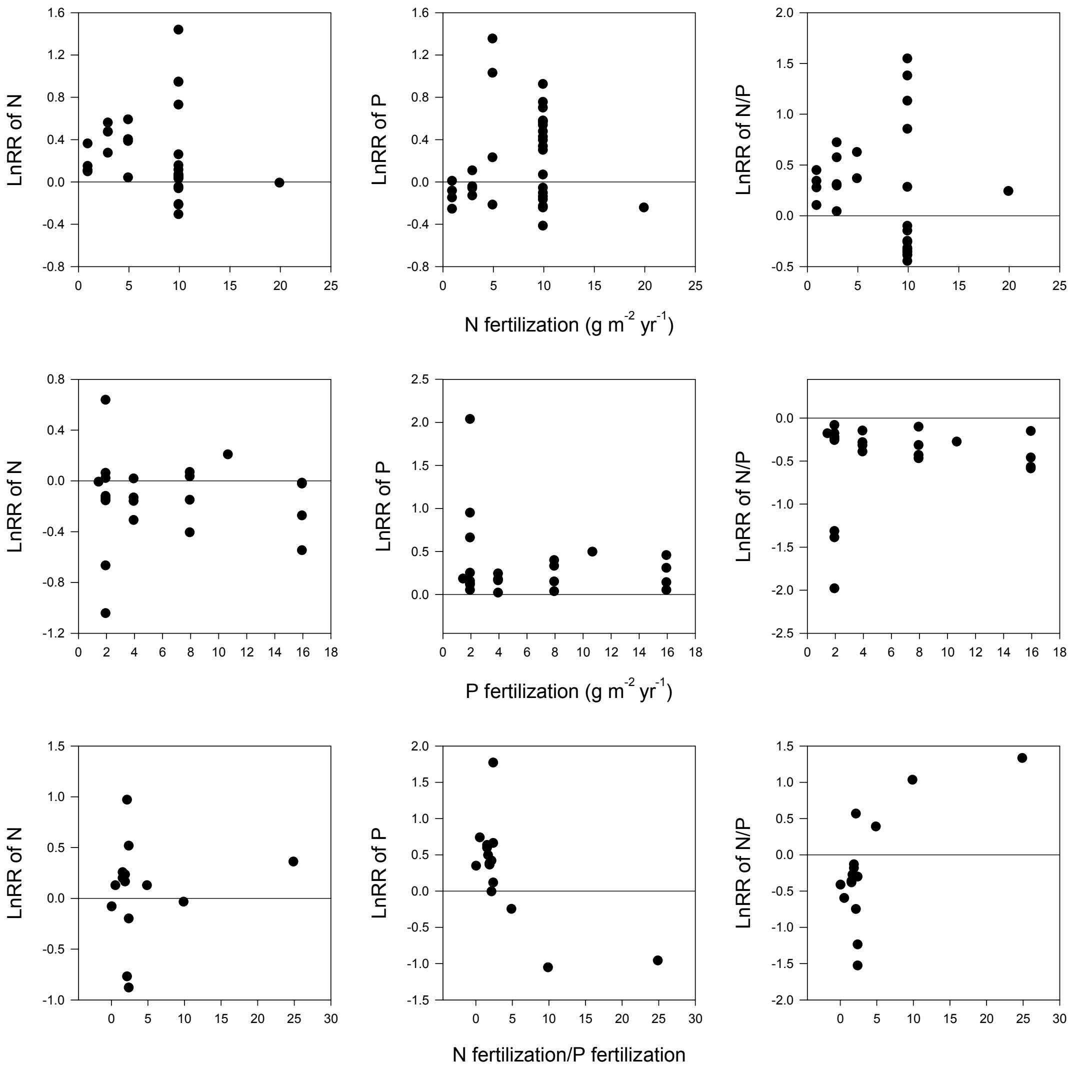

Figure S2. Relationships between nutrient fertilization and the response ratios of plant N concentrations, plant P concentrations and plant N/P to elevated CO_2 with nutrient fertilization. LnRR, the natural logarithm of response ratio that is calculated as the experimental mean divided by the control mean..

Supplementary references

Alberton O, Kuyper TW, Gorissen A. 2007. Competition for nitrogen between Pinus sylvestris and ectomycorrhizal fungi generates potential for negative feedback under elevated CO_2. *Plant and Soil* 296: 159-172.

Baxter R, Ashenden T, Farrar J. 1997. Effect of elevated CO_2 and nutrient status on growth, dry matter partitioning and nutrient content of Poa alpina var. vivipara L. *Journal of Experimental Botany* 48: 1477-1486.

Baxter R, Gantley M, Ashenden TW, Farrar JF. 1994. Effects of elevated carbon-dioxide on 3 grass species from montane pasture. 2. Nutrient-uptake, allocation and efficiency of use. *Journal of Experimental Botany* 45: 1267-1278.

Blank RR, Derner JD. 2004. Effects of CO_2 enrichment on plant-soil relationships of Lepidium latifolium. *Plant and Soil* 262: 159-167.

Brown ALP, Day FP, Hungate BA, Drake BG, Hinkle CR. 2007. Root biomass and nutrient dynamics in a scrub-oak ecosystem under the influence of elevated atmospheric CO_2. *Plant and Soil* 292: 219-232.

Brown K. 1991. Carbon dioxide enrichment accelerates the decline in nutrient status and relative growth rate of Populus tremuloides Michx. seedlings. *Tree Physiology* 8: 161-173.

Dijkstra FA, Pendall E, Morgan JA, Blumenthal DM, Carrillo Y, LeCain DR, Follett RF, Williams DG. 2012. Climate change alters stoichiometry of phosphorus and nitrogen in a semiarid grassland. *New Phytologist* 196: 807-815.

Duval BD, Dijkstra P, Drake BG, Johnson DW, Ketterer ME, Megonigal JP, Hungate

BA. 2013. Element pool changes within a scrub-oak ecosystem after 11 years of exposure to elevated CO_2. *PLoS ONE* 8: e64386.

Edwards EJ, McCaffery S, Evans JR. 2006. Phosphorus availability and elevated CO_2 affect biological nitrogen fixation and nutrient fluxes in a clover-dominated sward. *New Phytologist* 169: 157-167.

Finzi AC, Allen AS, DeLucia EH, Ellsworth DS, Schlesinger WH. 2001. Forest litter production, chemistry, and decomposition following two years of free-air CO_2 enrichment. *Ecology* 82: 470-484.

Finzi AC, Delucia EH, Schlesinger WH. 2004. Canopy N and P dynamics of a southeastern US pine forest under elevated CO_2. *Biogeochemistry* 69: 363-378.

Goverde M, Erhardt A, Stocklin J. 2004. Genotype-specific response of a lycaenid herbivore to elevated carbon dioxide and phosphorus availability in calcareous grassland. *Oecologia* 139: 383-391.

Hattas D, Stock WD, Mabusela WT, Green IR. 2005. Phytochemical changes in leaves of subtropical grasses and fynbos shrubs at elevated atmospheric CO_2 concentrations. *Global and Planetary Change* 47: 181-192.

Hou Y, Wang K-y, Zhang C. 2008. Effects of elevated CO_2 concentration and temperature on nutrient accumulation and allocation in Betula albo-sinensis seedlings. *The Journal of Applied Ecology* 19: 13-19.

Housman DC, Killingbeck KT, Evans RD, Charlet TN, Smith SD. 2012. Foliar nutrient resorption in two Mojave Desert shrubs exposed to Free-Air CO_2 Enrichment (FACE). *Journal of Arid Environments* 78: 26-32.

Hungate BA, Dijkstra P, Wu ZT, Duval BD, Day FP, Johnson DW, Megonigal JP, Brown ALP, Garland JL. 2013. Cumulative response of ecosystem carbon and nitrogen stocks to chronic CO_2 exposure in a subtropical oak woodland. *New Phytologist* 200: 753-766.

Jauhiainen J, Vasander H, Silvola J. 1998. Nutrient concentration in Sphagna at increased N-deposition rates and raised atmospheric CO_2 concentrations. *Plant Ecology* 138: 149-160.

Jin J, Tang C, Armstrong R, Sale P. 2012. Phosphorus supply enhances the response of legumes to elevated CO_2 (FACE) in a phosphorus-deficient vertisol. *Plant and Soil* 358: 86-99.

Johnson D, Ball J, Walker R. 1997. Effects of CO_2 and nitrogen fertilization on vegetation and soil nutrient content in juvenile ponderosa pine. *Plant and Soil* 190: 29-40.

Johnson D, Cheng W, Joslin J, Norby R, Edwards N, Todd D. 2004. Effects of elevated CO_2 on nutrient cycling in a sweetgum plantation. *Biogeochemistry* 69: 379-403.

Jongen M, Fay P, Jones MB. 1996. Effects of elevated carbon dioxide and arbuscular mycorrhizal infection on Trifolium repens. *New Phytologist* 132: 413-423.

Kanowski J. 2001. Effects of elevated CO_2 on the foliar chemistry of seedlings of two rainforest trees from north ‐ east Australia: Implications for folivorous marsupials. *Austral Ecology* 26: 165-172.

Kasurinen A, Riikonen J, Oksanen E, Vapaavuori E, Holopainen T. 2006. Chemical

composition and decomposition of silver birch leaf litter produced under elevated CO_2 and O_3. *Plant and soil* 282: 261-280.

Liu J, Huang W, Zhou G, Zhang D, Liu S, Li Y. 2013. Nitrogen to phosphorus ratios of tree species in response to elevated carbon dioxide and nitrogen addition in subtropical forests. *Global Change Biology* 19: 208-216.

Luomala EM, Laitinen K, Sutinen S, Kellomaki S, Vapaavuori E. 2005. Stomatal density, anatomy and nutrient concentrations of Scots pine needles are affected by elevated CO_2 and temperature. *Plant Cell and Environment* 28: 733-749.

Menge DNL, Field CB. 2007. Simulated global changes alter phosphorus demand in annual grassland. *Global Change Biology* 13: 2582-2591.

Murray MB, Smith RI, Friend A, Jarvis PG. 2000. Effect of elevated CO_2 and varying nutrient application rates on physiology and biomass accumulation of Sitka spruce (Picea sitchensis). *Tree Physiology* 20: 421-434.

Newbery R, Wolfenden J, Mansfield T, Harrison A. 1995. Nitrogen, phosphorus and potassium uptake and demand in Agrostis capillaris: the influence of elevated CO2 and nutrient supply. *New Phytologist* 130: 565-574.

Niinemets Ü, Tenhunen J, Canta N, Chaves M, Faria T, Pereira J, Reynolds J. 1999. Interactive effects of nitrogen and phosphorus on the acclimation potential of foliage photosynthetic properties of cork oak, Quercus suber, to elevated atmospheric CO_2 concentrations. *Global Change Biology* 5: 455-470.

Niklaus PA, Leadley PW, Stöcklin J, Körner C. 1998. Nutrient relations in calcareous grassland under elevated CO_2. *Oecologia* 116: 67-75.

Novotny AM, Schade JD, Hobbie SE, Kay AD, Kyle M, Reich PB, Elser JJ. 2007. Stoichiometric response of nitrogen-fixing and non-fixing dicots to manipulations of CO_2, nitrogen, and diversity. *Oecologia* 151: 687-696.

Olsrud M, Carlsson BÅ, Svensson BM, Michelsen A, Melillo JM. 2010. Responses of fungal root colonization, plant cover and leaf nutrients to long‐term exposure to elevated atmospheric CO_2 and warming in a subarctic birch forest understory. *Global Change Biology* 16: 1820-1829.

Pal M, Karthikeyapandian V, Jain V, Srivastava A, Raj A, Sengupta U. 2004. Biomass production and nutritional levels of berseem (Trifolium alexandrium) grown under elevated CO_2. *Agriculture, ecosystems & environment* 101: 31-38.

Peñuelas J, Filella I, Tognetti R. 2001. Leaf mineral concentrations of Erica arborea, Juniperus communis and Myrtus communis growing in the proximity of a natural CO_2 spring. *Global Change Biology* 7: 291-301.

Peñuelas J, Idso SB, Ribas A, Kimball BA. 1997. Effects of long‐term atmospheric CO2 enrichment on the mineral concentration of Citrus aurantium leaves. *New Phytologist* 135: 439-444.

Qiao YZ, Wang KY, Mang YB. 2007. Effects of elevated CO_2 on the growth and nutrient contents of Betula albosinensis seedlings with two planting densities. *Chinese Journal of Ecology* 26: 301-306.

Roberntz P, Stockfors J. 1998. Effects of elevated CO_2 concentration and nutrition on net photosynthesis, stomatal conductance and needle respiration of field-grown Norway spruce trees. *Tree physiology* 18: 233-241.

Rouhier H, Read D. 1998. Plant and fungal responses to elevated atmospheric carbon dioxide in mycorrhizal seedlings of *Pinus sylvestris*. *Environmental and Experimental Botany* 40: 237-246.

Rouhier H, Read DJ. 1999. Plant and fungal responses to elevated atmospheric CO_2 in mycorrhizal seedlings of *Betula pendula*. *Environmental and Experimental Botany* 42: 231-241.

Shinano T, Yamamoto T, Tawaraya K, Tadokoro M, Koike T, Osaki M. 2007. Effects of elevated atmospheric CO_2 concentration on the nutrient uptake characteristics of Japanese larch (Larix kaempferi). *Tree physiology* 27: 97-104.

Silvola J, Ahlholm U. 1996. Effects of CO_2 concentration on the nutrition of willows (Salix phylicifolia) grown at different nutrient levels in organic-rich soil. *Silva Fennica* 30: 221-228.

Watanabe M, Watanabe Y, Kitaoka S, Utsugi H, Kita K, Koike T. 2011. Growth and photosynthetic traits of hybrid larch F1 (*Larix gmelinii* var. *japonica*×*L. kaempferi*) under elevated CO_2 concentration with low nutrient availability. *Tree physiology* 31: 965-975.

Watling J, Press M. 1998. How does the C4 grass Eragrostis pilosa respond to elevated carbon dioxide and infection with the parasitic angiosperm Striga hermonthica? *New phytologist* 140: 667-675.

Whitehead S, Caporn S, Press M. 1997. Effects of elevated CO_2, nitrogen and phosphorus on the growth and photosynthesis of two upland perennials: Calluna vulgaris and Pteridium aquilinum. *New Phytologist* 135: 201-211.

Woodin S, Graham B, Killick A, Skiba U, Cresser M. 1992. Nutrient limitation of the long term response of heather [Calluna vulgaris (L.) Hull] to CO_2 enrichment. *New Phytologist* 122: 635-642.

Zhang S, Dang QL, Yü X. 2006. Nutrient and [CO_2] elevation had synergistic effects on biomass production but not on biomass allocation of white birch seedlings. *Forest ecology and management* 234: 238-244.
